# Platelet lysate gel and endothelial progenitors stimulate microvascular network formation *in vitro*: tissue engineering implications

**DOI:** 10.1038/srep25326

**Published:** 2016-05-04

**Authors:** Tiago M. Fortunato, Cristina Beltrami, Costanza Emanueli, Paul A. De Bank, Giordano Pula

**Affiliations:** 1 Department of Pharmacy and Pharmacology, University of Bath, Bath, UK; 2 Bristol Heart Institute, School of Clinical Sciences University of Bristol, Bristol, UK

## Abstract

Revascularisation is a key step for tissue regeneration and complete organ engineering. We describe the generation of human platelet lysate gel (hPLG), an extracellular matrix preparation from human platelets able to support the proliferation of endothelial colony forming cells (ECFCs) in 2D cultures and the formation of a complete microvascular network *in vitro* in 3D cultures. Existing extracellular matrix preparations require addition of high concentrations of recombinant growth factors and allow only limited formation of capillary-like structures. Additional advantages of our approach over existing extracellular matrices are the absence of any animal product in the composition hPLG and the possibility of obtaining hPLG from patients to generate homologous scaffolds for re-implantation. This discovery has the potential to accelerate the development of regenerative medicine applications based on implantation of microvascular networks expanded *ex vivo* or the generation of fully vascularised organs.

Insufficient vascularization is not only the main cause of chronic ischaemic diseases but also the main bottleneck towards the development of tissue-engineered grafts for clinical use^1^. The shortage of adequate extracellular matrices that can support blood vessel formation/regeneration is a major limitation in regenerative medicine and tissue engineering^2^. The combined delivery of angiogenic growth factors (GFs) and endothelial progenitor cells (EPCs) within a supporting scaffold has been suggested as an approach capable of solving this challenge^3^.

Endothelial Colony Forming Cells (ECFCs) are a subtype of EPCs that can be isolated from circulating precursors in human peripheral blood. They have been suggested as a viable source of autologous cells for therapeutic vasculogenesis^4^. For the formation of functional and stable new blood vessels, it is crucial that different angiogenic signals cooperate to stimulate the formation of new tubular structures^5^. Therapeutic approaches based on the delivery of a single GF, such as vascular endothelial growth factor (VEGF), usually fail to promote the formation of a healthy and lasting vasculature^6^,^7^ due to inefficient recruitment of perivascular cells and endothelial cells (ECs)^5^. Growing evidence shows that ECs respond efficiently to matrix-bound GFs. This may be due to the establishment of a spatial GF gradient that cells can sense and migrate towards, and to the crosstalk between integrins and GF receptors^6^,^8^,^9^,^10^. While synthetic polymers have been extensively investigated for the delivery of GFs due to their off-the-shelf nature, their use is often dependent on the delivery of recombinant GFs and suffers the same limitations in terms of efficiency, safety and cost^11^. Moreover, despite a few very recent examples^12^, synthetic polymers do not adhere efficiently to the target tissue and sometimes lack the ideal degradation profile. On the other hand, animal-derived biomaterials can naturally overcome these issues but their approval for clinical applications can be problematic due to the risk of animal-borne disease transmission to the recipients and other adverse reactions to animal products^13^,^14^.

Platelets are a natural reservoir of angiogenic GFs and are activated at sites of tissue injury, where they promote tissue repair, including revascularization^15^. Therefore, through lysis of human platelets, GFs can be extracted, providing a less expensive and safer alternative to the use of recombinant or animal products^16^. Human platelet lysate (hPL) as a source of supplements for cell culture has been described^17^,^18^, including its application as culture medium supplement for the culture of endothelial progenitor cells^19^. In addition, platelet-enriched plasma (PRP) also contains extracellular matrix (ECM) precursors, like fibrinogen, offering a fully human option for a biodegradable scaffold. Fibrinogen can be quickly polymerized into fibrin at body temperature through the coagulation cascade and further bind to other scaffolding proteins present in plasma, such as fibronectin, or originating from platelets, like vitronectin^20^. These proteins are adhesive to collagen and other ECM components in injury sites and contain integrin-binding sites (e.g. RGD motifs), allowing cell attachment. Importantly, the backbone of platelet-derived scaffolds can be gradually degraded by the action of proteases released by cells, such as plasmin and matrix metalloproteinases (MMPs), therefore achieving full biodegradability and host integration^21^.

In this study, we investigated the use of human platelets as a source of GFs and structural proteins for the generation of a complete 3D cell culture support. Previous studies on human stem cells demonstrated the possibility of fabricating 3D cell culture scaffolds using human platelet lysates^22^. Nonetheless, this approach has never been utilized for the culture of EPCs and, most importantly, the ability of human platelet lysate gel (hPLG) to support the formation of vascular capillaries by EPCs has never been investigated before. Here, we describe the use of hPLG as an animal product-free and patient-specific tool to amplify, differentiate and deliver ECFCs for regenerative medicine applications. We characterized the structure and composition of hPLG. We then demonstrated the ability of this human-derived material to promote ECFC amplification and stimulate the formation of ECFC-driven microvascular networks *in vitro*. Finally, we characterized the morphology and properties of the microvascular networks obtained *in vitro*. These observations will open the way to the utilization of hPLG to support the development of ECFC-based cell treatments for human health.

## Results

### hPLG contains and releases multiple angiogenic GFs

**Figure 1:**
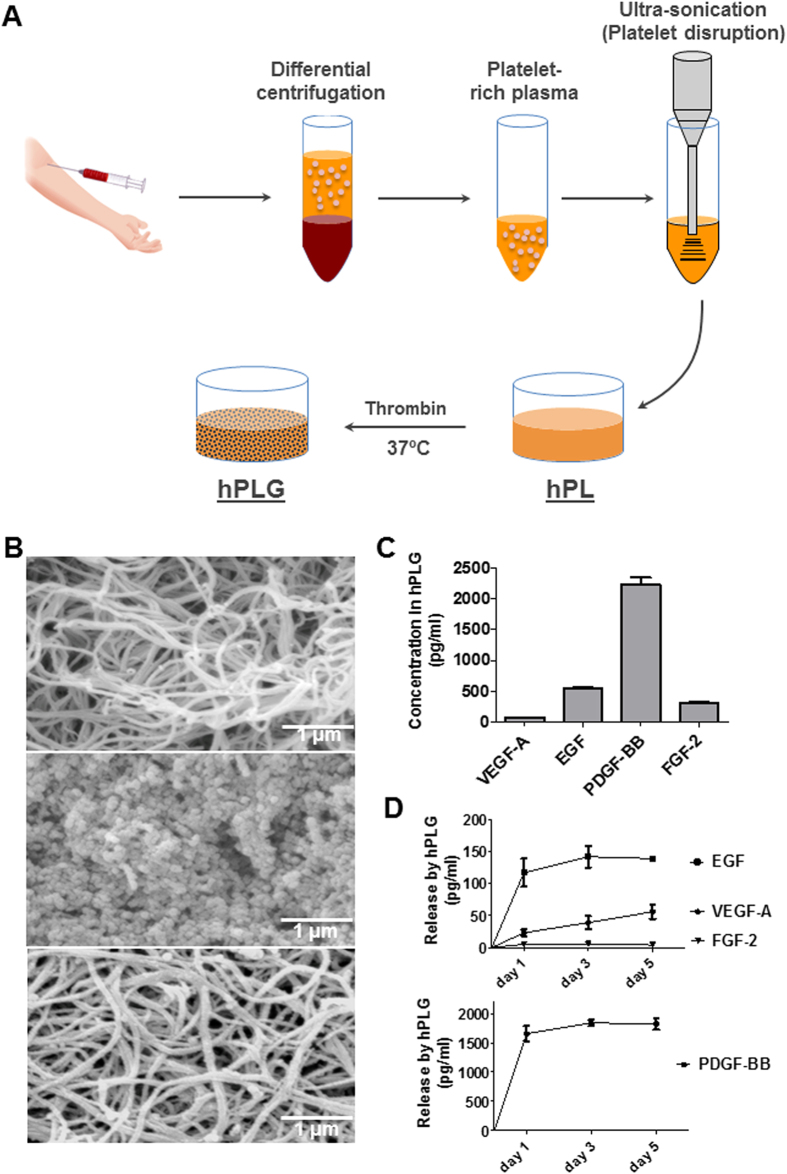
Production and characterization of hPLG. (**A**) Platelet-rich plasma obtained from whole blood or apheresis is by ultrasonication and gelled at 37 °C by adding human thrombin. (**B**) Scanning electron microscopy (SEM) images of fresh fibrin (top), fresh hPLG (middle) and hPLG after 3 days incubation (bottom) at 20,000x magnification. (**C**,**D**) Initial concentration and release of GFs in 1, 3 or 5 days of culture by hPLG were determined by ELISA. Data show mean ± SEM. Data are representative of three independent experiments.

The protocol for the preparation of hPLG is described in Fig. 1A and entails: platelet rich plasma (PRP) isolation from peripheral whole blood, its ultrasonication to disrupt the cellular fraction (i.e. platelets) and its gelation using thrombin. The structure of hPLG has been investigated by scanning electron microscopy (SEM) in Fig. 1B, which revealed a fibrous matrix very similar to fibrin gels (top panel) with an abundant protein coating (mid panel). The protein coating appears to be released by hPLG through time, as at day 3 the protein coating has largely, yet not completely, disappeared (bottom panel). We also determined the identity and concentrations of GFs in hPLG (Fig. 1C). ELISA experiments showed that hPLG before gelation contains amounts of VEGF-A (77 ± 50 pg/mL), EGF (545 ± 49 pg/mL), PDGF-BB (2,293 ± 20 pg/mL) and FGF-2 (318 ± 10 pg/mL), comparable with the previously reported values in literature for platelet lysates produced by freeze-thaw methods^23^,^24^,^25^. We then analyzed the release of these GFs by hPLG in culture medium. Only VEGF-A, EGF and PDGF-BB appeared to be efficiently released, with only the latter reaching biologically-relevant concentrations above 1 ng/mL (Fig. 1D).

### Culture of ECFCs on hPLG stimulates cell proliferation in a VEGF-dependent manner

**Figure 2:**
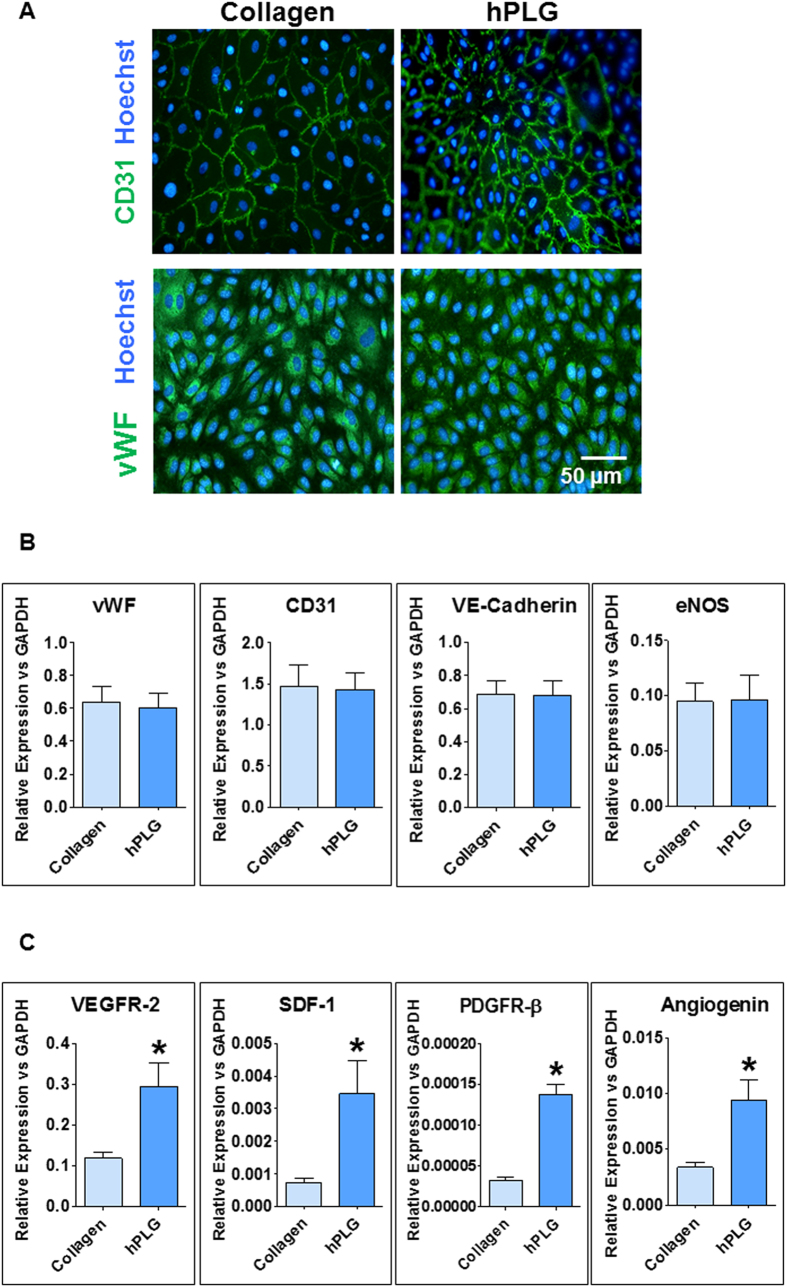
hPLG as a support for culture of ECFCs. (**A**) ECFC morphology was confirmed by immunofluorescence for specific endothelial markers CD31 and vWF on collagen-coated plastic and hPLG. Total RNA was extracted after 24 h of ECFCs seeding on collagen-coated plastic and hPLG. Expression of endothelial (**B**) and pro-angiogenic (**C**) marker genes was assessed by RT-qPCR. The 2^−ΔΔCt^ analysis method was used to analyze the data with GAPDH used as normaliser. Data show mean ± SEM and are representative of three independent experiments. Statistical significance was tested using Mann-Whitney U-test (*P ≤ 0.05).

**Figure 3:**
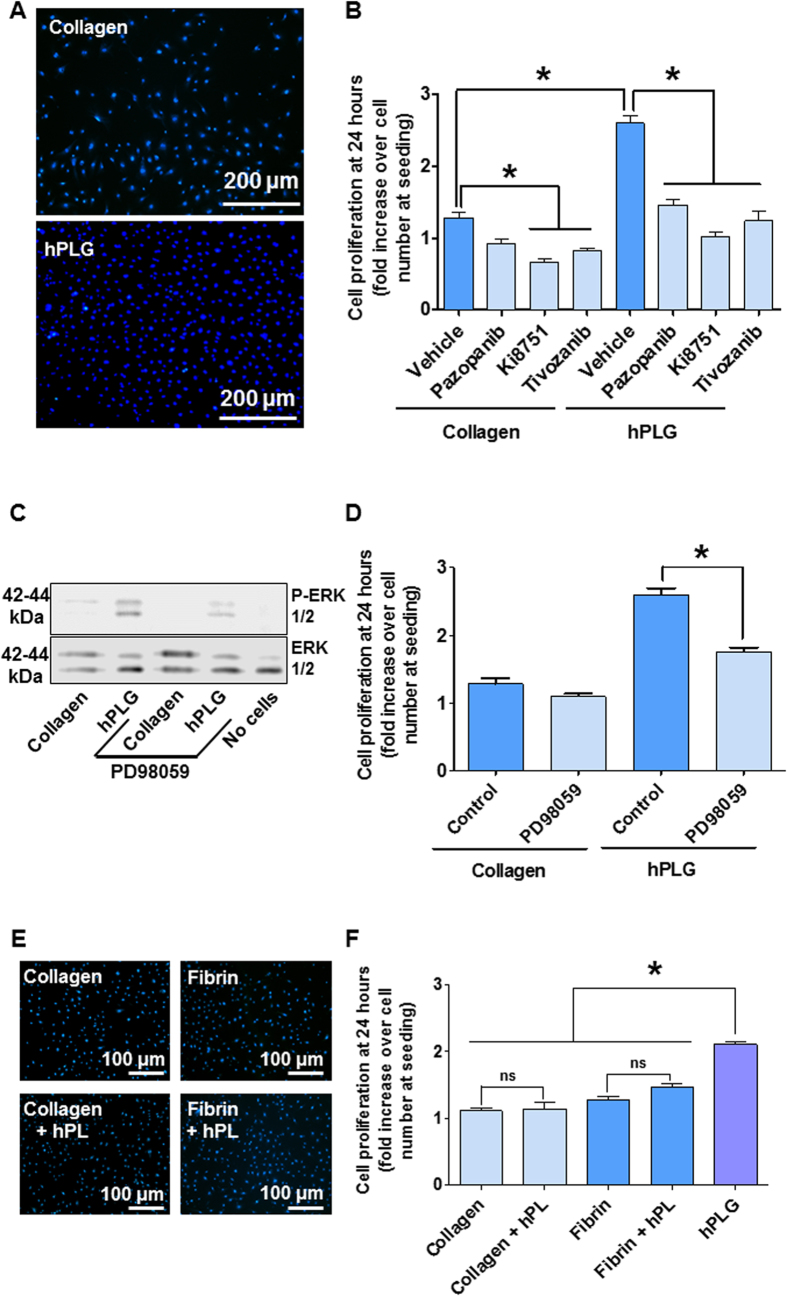
hPLG stimulates ECFC proliferation. (**A**) 12,000 ECFCs/well were seeded on collagen-coated plastic and hPLG and cultured for 24 h in complete culture medium before being PFA-fixed and nuclear-stained with Hoechst 33342 (in blue). (**B**) ECFCs were cultured either on rat collagen I or hPLG and incubated with Pazopanib (20 μg/mL), Ki8751 (100 nM) or Tivozanib (1 μM). The number of cell per mm^2^ was then estimated by microscopy. Data show mean ± SEM and are representative of three independent experiments. Statistical significance was tested by one-way ANOVA with Bonferroni’s post-test to compare different inhibitors against the control conditions (*P ≤ 0.05). (**C**) Activation of ERK1/2 by contact with hPLG versus rat collagen I coating (incubation 1 hour in complete medium) and its inhibition by 50 μM PD98059. ERK1/2 phosphorylation was tested by phospho-specific immunoblotting. Last lane demonstrates expression but no phosphorylation of ERK1/2 from platelets in hPLG (in the absence of ECFCs). (**D**) ERK inhibition by 50 μM PD98059 significantly inhibits ECFC proliferation on hPLG but not on rat collagen I coating. Data show mean ± SEM and are representative of six independent experiments. ECFC proliferation was also tested in 2D cultures on collagen and fibrin gels with hPL provided as a source of extra GFs. Representative pictures are shown in (**E**) and quantitative analysis is shown in (**F**). As normal distribution and equal variance of the samples were confirmed using the Shapiro-Wilk normality test and Bartlett’s homoscedasticity test (Supplementary Table 2), statistical significance was tested using one way ANOVA with Bonferroni post-test (*P ≤ 0.05, n = 6).

The suitability of hPLG as a substitute for traditional surface coating for the maintenance of ECFC cultures (i.e. rat collagen I) was confirmed by immunofluorescence for endothelial markers CD31 and von Willebrand Factor (vWF) (Fig. 2A). The phenotype of ECFCs cultured on hPLG was also confirmed by qPCR for a series of endothelial markers: VE-cadherin, endothelial nitric oxide synthase (eNOS), CD31 and vWF (Fig. 2B). Interestingly, expression of a series of pro-angiogenic molecules such as VEGF receptor 2 (VEGFR2), platelet-derived growth factor receptor-β (PDGFR-β), angiogenin and stromal-derived factor 1 (SDF-1) was significantly increased in ECFCs cultured on hPLG compared to collagen (Fig. 2C). We also observed a significantly higher proliferative potential of ECFCs cultured on hPLG compared to collagen. As shown in Fig. 3A by nuclear staining using Hoechst 33342 and epifluorescence imaging, the proliferation of ECFCs on hPLG was significantly higher than on collagen over a period of 24 hours (Fig. 3B). In addition, the role of GF signaling in the proliferation of ECFCs on hPLG was tested. VEGF receptor-specific Ki8751 and tivozanib significantly inhibited ECFC proliferation on both collagen and hPLG, whereas the less specific inhibitor Pazopanib only inhibits proliferation on hPLG (Fig. 3A,B). As ECFC proliferation on hPLG was completely abolished by VEGF inhibitors, we can conclude that similarly to what has been observed for cultures on collagen^26^, VEGF signaling is necessary for ECFC proliferation on hPLG. As expected, due to its role in VEGF and other GFs’ signaling, extracellular regulated kinase 1 and 2 (ERK1/2) appear to be robustly activated in ECFCs cultured on hPLG but not on collagen (Fig. 3C). The role of ERK1/2 activation in the proliferation of ECFCs was confirmed using an inhibitor of these kinases (PD98058), which inhibited proliferation on hPLG but not on collagen (Fig. 3D). In order to determine whether the GFs (e.g. VEGF) contained in hPL were responsible for the high proliferation observed on hPLG, we performed experiments with hPL utilized as liquid supplement for the culture medium (Fig. 3E,F). These experiments showed that the addition of the same quantity of liquid hPL per well as that used for the generation of hPLG resulted in significantly lower proliferation in comparison to ECFCs growing on hPLG. This suggests that the pro-proliferative activity of hPLG is not simply due to the presence/release of GFs.

### 3D culture of ECFCs in hPLG promotes capillary vascular network formation

**Figure 4:**
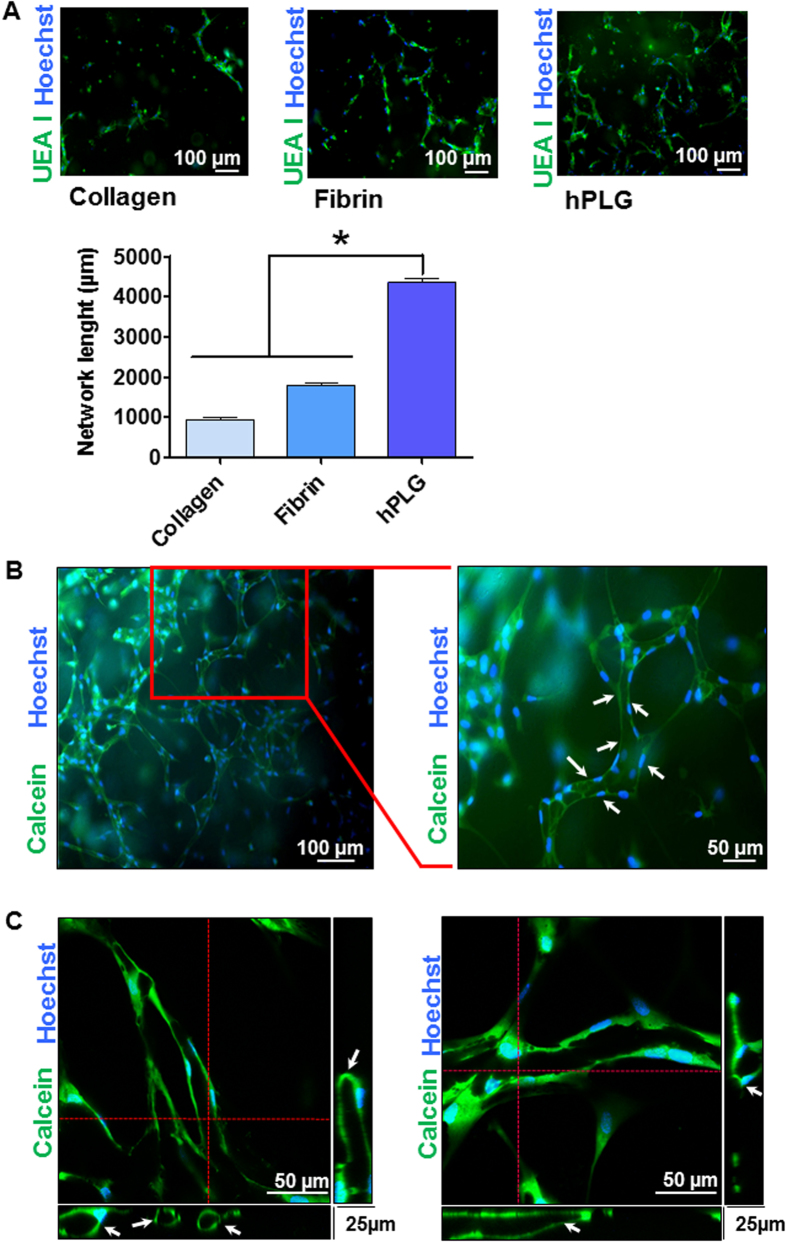
Detection of a lumenized vascular network by hPLG encapsulated ECFCs using epifluorescence and confocal microscopy. (**A**) ECFCs were encapsulated in collagen I, fibrin or hPLG and cultured for 3 days at 37 °C. Constructs were fixed and stained with FITC-conjugated Ulex europaeus lectin (FITC-UEA I) which binds to α-L-fucose-containing glycocompounds on the surface of endothelial cells and Hoechst 33342 to localize cell nuclei. Total length of the capillary network in the different conditions was measured using Angiogenesis Analyzer plugin of ImageJ 1.49 v. As normal distribution and equal variance of the samples were confirmed using the Shapiro-Wilk normality test and Bartlett’s homoscedasticity test (Supplementary Table 2), statistical significance was tested using one way ANOVA with Bonferroni post-test (*P ≤ 0.05, n = 6). hPLG-encapsulated ECFCs were live stained after 3 days of the start of the experiment. Fluorescent calcein AM was used to stain the cytoplasm of viable cells, while Hoechst 33342 was utilized to localize cell nuclei. Images show multiple ECFCs organized into primitive vascular structures with a lumen (white arrows). Epifluorescence images were obtained using a Leica DMI4000B microscope (objective: N Plan 10X/0.25) (**B**), whereas confocal microscopy was performed with a LSM 510 META confocal microscope (objective: EC Plan Neofluar 10X/0.30 M27) (**C**).

**Figure 5:**
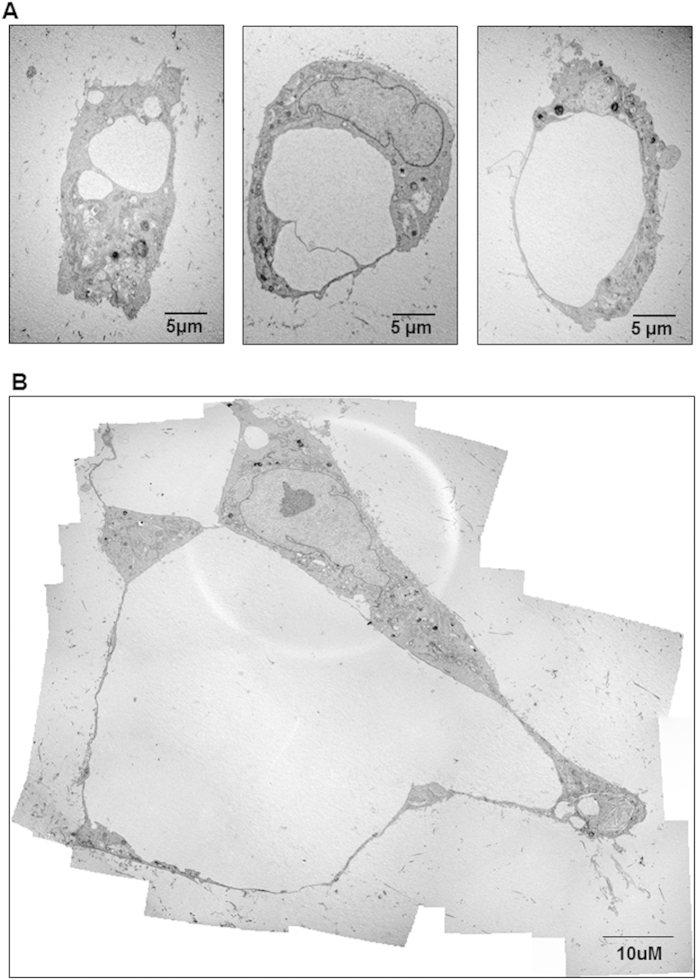
Detection of a lumenized vascular network by hPLG encapsulated ECFCs using transmission electron microscopy (TEM). TEM was used to assess whether the tubular structures developing in ECFCs cultured in hPLG have a lumen and display capillary morphology. Different stages of lumen formation are shown: from vacuole formation to lumens delimited by single cells (**A**) to multicellular lumen delimitation (**B**). Images are representative of three independent experiments.

**Figure 6:**
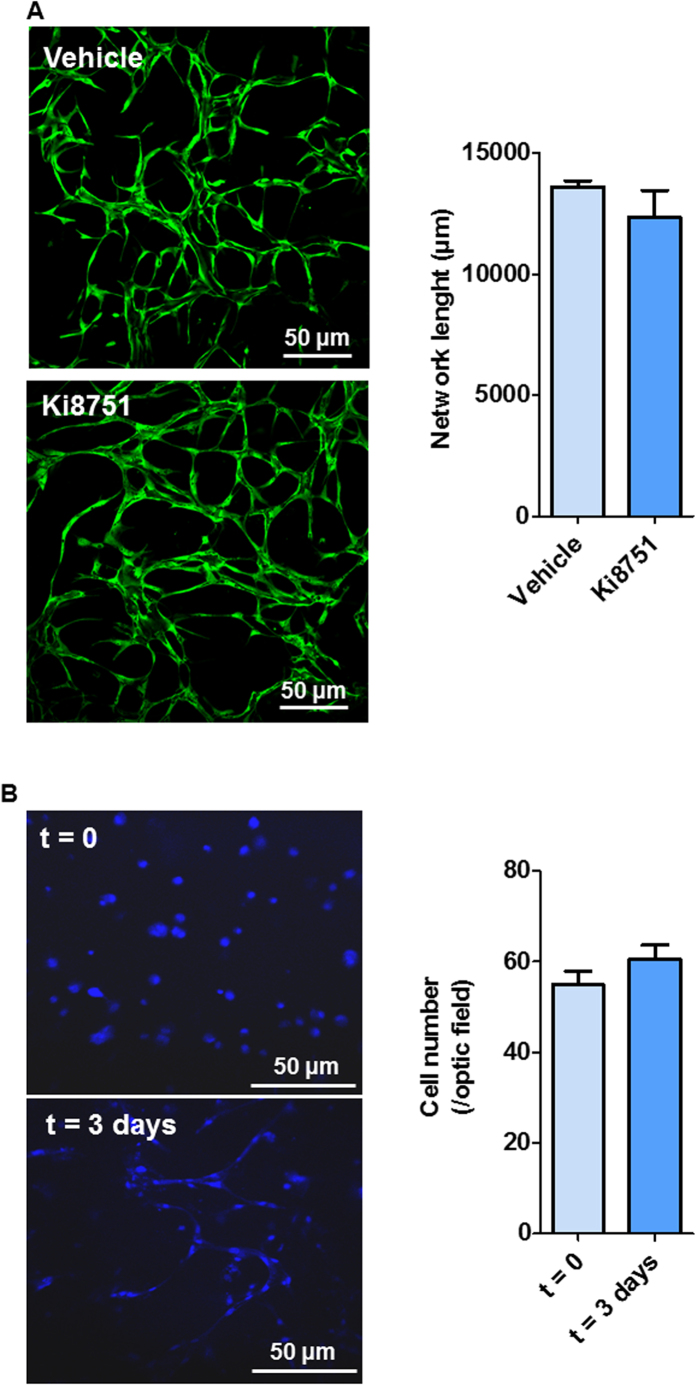
hPLG support capillary tube formation by encapsulated ECFCs in a VEGFR2- and proliferation-independent manner. ECFCs were embedded in hPLG as described and treated with Ki8751 (100 nM) or vehicle solution. After 24 hours, in order to assess the capillary tube formation (**A**), cells were stained with Calcein AM (10 μM, 1 h) and imaged by epifluorescence. Total length of the capillary network in the different conditions was measured using ImageJ 1.49 v. In order to assess ECFC proliferation in hPLG-based 3D cultures (**B**), cells were stained with Hoechst 33342 (1 μg/mL, 15 min) 72 hours after seeding and imaged by confocal microscopy. Number of nuclei was counted to estimate cell numbers. Data show mean ± SEM and are representative of three independent experiments. Statistical significance was tested using Mann-Whitney U-test (*P ≤ 0.05).

We investigated the potential of hPLG as a supportive scaffold for the formation of fully human capillary networks. To achieve this, ECFCs were encapsulated in hPLG and their behaviour was compared with ECFCs encapsulated in collagen I and fibrin gels. Cells in hPLG were cultured in serum-free basal medium, while complete medium (i.e. 10% FBS plus recombinant GFs) was added to cells in collagen or fibrin gels in order to ensure cell viability and angiogenic response (which cannot be achieved without FBS supplementation in collagen or fibrin gels). Over a 3 day period, ECFCs within hPLG established a *de novo* endothelial network as evidenced by dual staining with FITC-conjugated *Ulex europaeus* lectin (FITC-UEA I) and Hoechst 33342 (Fig. 4A). Conversely, there was negligible formation of an interconnected capillary network within collagen I or fibrin gels, as shown by capillary network formation quantitative analysis (Fig. 4A). The cell-permeant dye calcein AM was then used to clearly distinguish lumens lined by endothelial cells as part of the capillary structures in hPLG. Both epifluorescence (Fig. 4B) and confocal microscopy were used (Fig. 4C). Confocal images are shown with orthogonal views obtained by z-stacking, which clearly shows the presence of a lumen in the capillaries. The presence capillaries with a lumen within the ECFC cultured in hPLG was also confirmed by transmission electron microscopy (TEM) (Fig. 5A). Although the lumens of the capillaries reached a considerable size, due to limitations in TEM technique and data analysis, only capillaries with lumen <30 μm can be captured in one scan. Larger capillaries can be detected and displayed by building a composite picture made of several scans. One example shown here had a diameter of around 100 μm (Fig. 5B). The role of VEGFR2 in capillary tube formation in 3D cultures was investigated using the specific inhibitor Ki8751, which had no significant effect (Fig. 6A). Interestingly, ECFC proliferation was also negligible when cells were encapsulated in either hPLG (Fig. 6B) or other supports (data not shown). Taken together, these two observations suggest that cell proliferation does not significantly contribute to capillary formation in 3D cultures of ECFCs.

**Figure 7:**
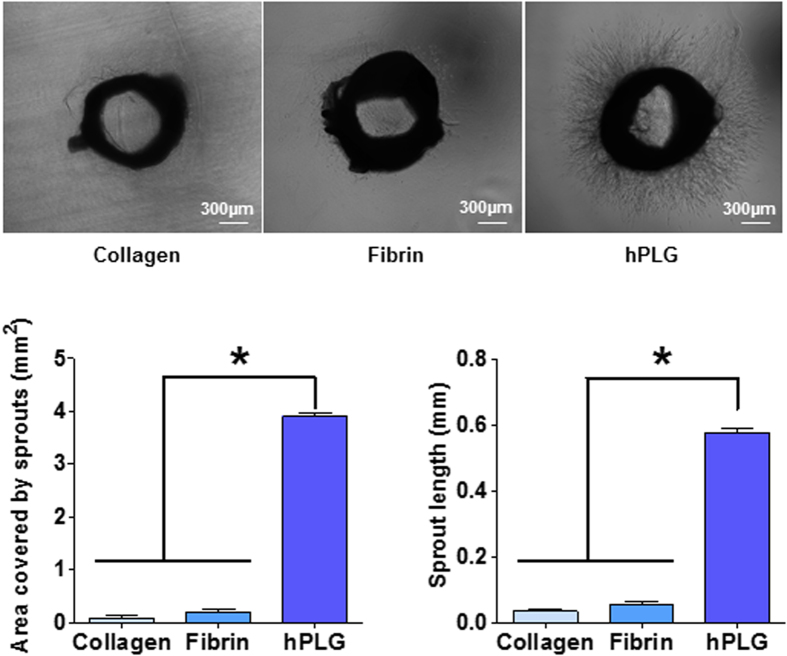
hPLG supports aortic ring sprouting. Cross sections of rat aorta were encapsulated in collagen, fibrin and hPLG and cultured in serum-free basal medium for 3 days at 37 °C. Aortic rings were fixed and images captured with an EVOS FL microscope Representative example are shown in (**A**). Angiogenesis was quantified as total sprouting area and average length of sprouts (**B**). Data show mean ± SEM. As normal distribution and equal variance of the samples were confirmed using the Shapiro-Wilk normality test and Bartlett’s homoscedasticity test (Supplementary Table 2), statistical significance was tested using one way ANOVA with Bonferroni post-test (*P ≤ 0.05, n = 6).

**Figure 8:**
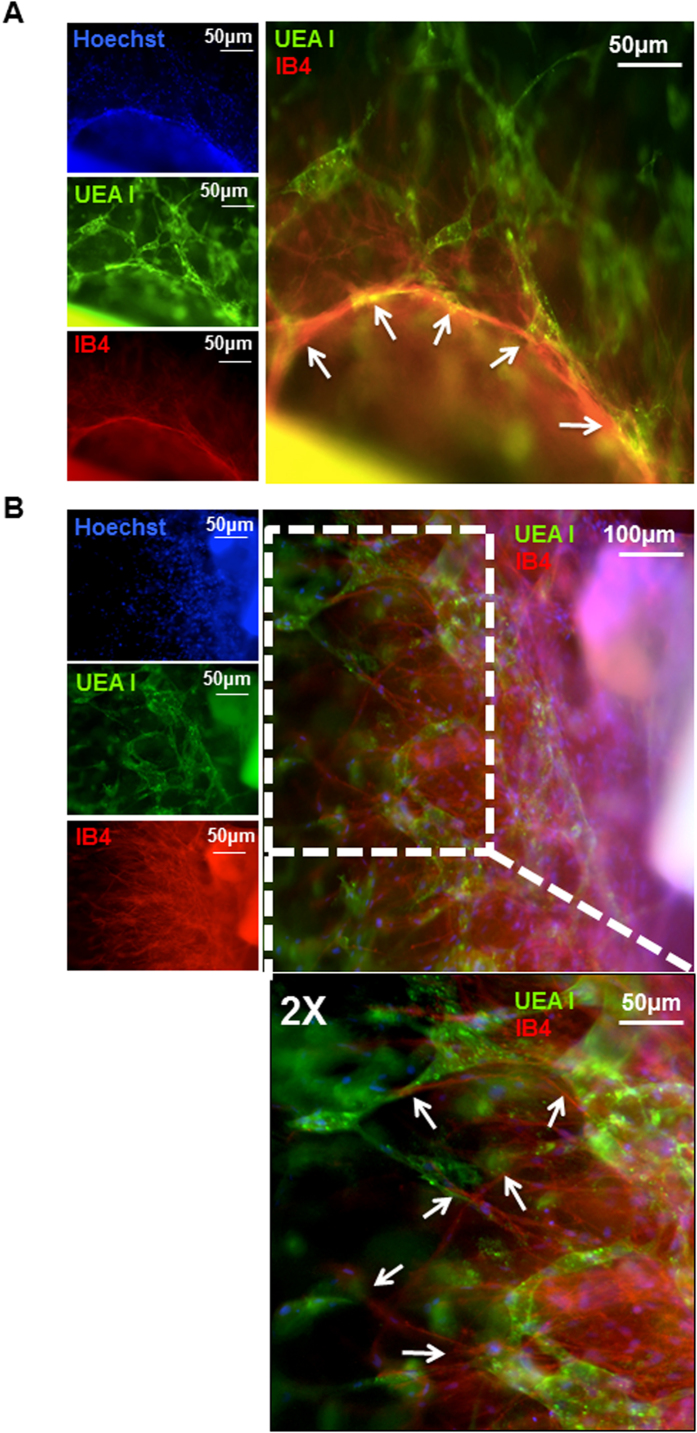
hPLG-induced ECFC capillary network interacts with rat aortic angiogenic sprouts. Cross sections of rat aorta were encapsulated in hPLG and cultured in serum-free basal medium, which led to robust formation of angiogenic sprouts. (**A**,**B)** display examples of co-culture of rat aortic ring and human ECFCs in hPLG. 3 days after encapsulation, gels were fixed and stained with FITC-conjugated Ulex europaeus lectin (FITC-UEA I) to specifically label human endothelial cells (ECs) and with TRITC-conjugated Isolectin B4 (IB4) which binds terminal α-galactosyl residues expressed on non-primate ECs, in addition to Hoechst 33342 which was used as nuclear staining. White arrows indicate sites of close between rat ECs and network formed by human ECFCs.

Next, we tested the ability of hPLG to support capillary network formation by different cell types and promote their interaction. Aortic ring sections from rat were co-cultured with ECFCs in hPLG to obtain angiogenic sprouting. Remarkably, hPLG supported a vigorous sprouting from rat aortic rings, whereas embedding in collagen or fibrin did not (Fig. 7). Next we assessed whether the capillary tube network from ECFCs interacted with the sprouts from the aortic tissue. Rat aortic sprouts were stained with TRITC-conjugated Isolectin B4 (IB4), which binds terminal α-galactosyl residues expressed on non-primate ECs. Human ECFCs were stained with FITC-UEA I. We identified points of alignment and interaction between human and rat vasculature (Fig. 8). This observation suggests that hPLG may provide a favorable environment for the integration of vascular networks of different origin.

## Discussion

The delivery of EPCs and angiogenic GFs has been proposed as a viable approach for vascularization of tissue-engineered grafts as well as the treatment of ischaemic conditions^3^,^6^,^27^,^28^,^29^. Here, we investigated the use of hPLG as a potential injectable scaffold that is enriched in several angiogenic GFs to expand and deliver human ECFCs for therapeutic applications. We focused on the use of hPLG for amplification and differentiation into endothelial capillary networks of ECFCs. The standard isolation protocol for ECFCs initially developed by Ingram *et al.*^30^ was utilized to isolate ECFCs in this study. In agreement with a previous study^31^, platelet lysate preparations added to the culture medium as a supplement provide a source of human GFs and allow ECFC culture in complete absence of animal products.

To obtain a human tissue-based scaffold enriched in platelet-derived GFs, we have opted for lysing platelet-rich plasma by ultrasonication. This method disrupts platelets and their alpha-granules through cavitation, diminishing the possible negative impact on GF activity of repeated freeze-thaw methods^32^. In this study we show that hPL contains significant amounts of VEGF-A, EGF, PDGF-BB and FGF-2 (Fig. 1C). This panel is further complemented by ANGPT-1, IGF-1, PDGF-AB and TGFβ-1, as previously demonstrated^23^,^24^,^25^. These GFs have been shown to play a role in tissue repair through promotion of angiogenesis, due to their chemotactic, mitogenic and anti-apoptotic effects on both endothelial cells and perivascular cells (pericytes and smooth muscle cells)^33^,^34^. As expected from the concentrations of GFs measured in liquid hPL, when entrapped within the matrix of hPLG, only PDGF-BB release reached biologically-relevant concentrations above 1 ng/mL when studied over 5 days (Fig. 1D). The release of VEGF-A from hPLG (<100 pg/mL after 5 days) was significantly lower than the concentration in complete culture medium (2 ng/mL). This suggests that the effects of hPLG on ECFC culture cannot be simply due to the release of GFs. It seems plausible to suggest that the ECM proteins and other adhesive substrates in hPLG facilitate integrin interactions and sequester GFs, resulting in higher cell adhesion, proliferation and vasculogenic activity^35^. The presentation of GFs as ECM-bound rather than in solution may also provide an important boost to cell proliferation^6^,^8^,^36^.

The importance of VEGF in the stimulation of ECFC proliferation has been suggested by this study. In particular, the activation of VEGFR2 appears to play a role in the stimulation of ECFC proliferation cultured either on hPLG- or collagen-coated surfaces (2D culture shown in Fig. 3A). This was demonstrated by the effects of the VEGF-specific inhibitors Ki8751 and tivozanib. These data are in agreement with previous literature^26^ and, although they do not clarify the molecular mechanism underlying increased ECFC proliferation on hPLG, suggest that VEGFR2 signalling is necessary for the phenomenon to take place. Our ELISA data suggested that the amount of VEGF directly released by hPLG to the culture is significantly lower than the concentration provided in the complete cell culture medium and is therefore unlikely to cause increased ECFC proliferation. It seems plausible to assume that the increased expression of VEGFR2 highlighted by our qPCR experiments (Fig. 2C) renders ECFCs more responsive to VEGF and induces a VEGF-dependent surge in cell proliferation. Another possibility is that the presentation of ECM-bound GFs generates protracted and more effective signaling on target cells, when compared to liquid-phase GFs^8^,^9^,^10^,^36^. The proximity of GF-binding domains and integrin-binding sites in ECM proteins may facilitate the formation of focal adhesion complexes and regulate the activity of associated GF receptors^37^,^38^. For example, VEGF signaling in endothelial cells is impaired when the binding of αvβ3 integrin to RGD motifs in vitronectin is blocked^39^ and angiogenesis regulated by FGF-2 is dependent on binding of integrin α5β1 to fibronectin^40^.

In contrast to proliferation data in 2D cultures, the formation of vascular structures in 3D ECFC culture in hPLG is only marginally affected by VEGFR inhibition (Fig. 6A)., This suggests that other characteristics of hPLG play a key role in the 3D organisation of ECFCs to form capillaries, such as ECM-bound adhesion molecule substrate presentation. One credible explanation to this observation is that ECFC proliferation is marginal when cells are embedded in hPLG (as shown in our data in Fig. 6B). Therefore, the increase in cell proliferation driven by the VEGF/VEGFR2 axis has no outcome in terms of capillary formation. It is important to highlight here that the delivery of high amounts of recombinant human VEGF (rhVEGF) has been extensively attempted to promote *in situ* angiogenesis/vasculogenesis. It is now known that this leads to aberrant blood vessel formation^7^,^41^. More recently, the combined delivery of VEGF and PDGF-BB has been shown to be necessary to induce normal angiogenesis^42^. By using hPLG, we can deliver both these GFs, among others, and induce more efficiently the formation of capillary vascular networks. In addition to the presence of multiple GFs, it is likely that the hPLG-driven responses in 3D cultures (i.e. capillary network formation) depend on the effect of adhesive substrates presented to the ECFCs by hPLG.

Interestingly, we report for the first time a coordinated upregulation of a set of genes involved in angiogenesis by endothelial cells in response to a biomaterial. The expression of VEGFR2, SDF-1, angiogenin and PDGFR-β by ECFCs was significantly upregulated on hPLG after 24 h (Fig. 2C). One likely consequence of this is an increased sensitivity of ECFCs to VEGF and PDGF in this culture conditions. One plausible hypothesis is that binding of different integrins to fibrin and other ECM proteins in hPLG may initiate a cascade of events leading to an increase in the transcription levels of these receptors. Indeed, it is known that the attachment to RGD motifs via integrins αvβ3 and α5β1 is required for endothelial morphogenesis in fibrin matrices in the presence of high concentrations of GFs^43^. The combination of GF supply, pro-angiogenic ECM protein presentation and tridimensional features of hPLG supports the formation of a profuse microvascular network by human ECFCs. The tridimensional vascular network formed *in vitro* was not only more complete than those formed in collagen I and fibrin gels (Fig. 4A), but also exhibited a well-defined lumen generated in a period of just 3 days (Fig. 4C and Fig. 5). Therefore, hPLG is ideally suited for the *in vivo* delivery of ECFCs and possibly other pro-angiogenic cells. It also opens the way to very promising clinical applications: 1) the stimulation of tissue repair by *in vitro* formation of microvascular networks followed by their implantation or injection in patients; 2) the engineering of complete organs equipped with a functional microvascular networks.

In conclusion, we have shown that hPLG strongly promotes the proliferation of ECFCs in 2D cultures in a VEGF- and ERK-dependent manner. Although the underlying mechanisms remain to be fully elucidated, the GF content of hPLG does not explain this phenomenon as hPLG greatly exceeds the efficiency of hPL (as a liquid media supplement) in stimulating ECFC proliferation. The upregulation of a series of angiogenic genes (including VEGFR2) is the likely cause for the increased proliferation of ECFCs in 2D cultures on hPLG. Within 3D cultures (i.e. cell encapsulation in hPLG), the biochemical environment provided by hPLG induces vasculogenic responses leading to the formation of an extensive capillary network by ECFCs. We propose the use of hPLG as a human-derived expansion and delivery tool for clinical uses of ECFCs.

## Methods

### Preparation of human platelet lysate gel (hPLG), rat collagen I gels and human fibrin gels

Bags of leukocyte-depleted platelet-rich plasma (PRP) prepared by cytapheresis were kindly provided by the Royal United Hospitals (RUH) Bath NHS Foundation Trust. The bags were anonymised and released for research purposes under the ethical approval of the South West - Central Bristol Research Ethics Committee (REC) after their expiry. Preparation and handling were performed in accordance with the guidelines approved by the Ethics Committee of the University of Bath. The platelet count for each unit was >2.40 × 10^11^ in an average volume of 200 mL plasma supplemented with citrate to prevent platelet activation. Platelets from 5 or more donors were pooled and lysed by ultrasonication using a Model 150VT Ultrasonic Homogenizer (BioLogics, Manassas, VA, USA). Aliquots were then centrifuged at 4,000× *g* (15 min, 4 °C) to remove platelet fragments and filtered through a 0.2 μm pore filter to obtain human platelet lysate (hPL). To induce gelation, hPL was diluted in basal medium (20% v/v) (EBM-2, Lonza, Walkersville, US) and human thrombin (Sigma-Aldrich, Poole, UK) was added to a final concentration of 0.5 U/mL. Formation of hPLG (i.e. gelation) was observed within 5 minutes from thrombin addition. Following a wash with phosphate buffer saline (PBS).

For collagen gel coating, rat-tail collagen I (Corning) was diluted to a final concentration of 1.5 mg/mL and polymerized by neutralizing the pH using 1 M NaOH, as previously described^44^. For fibrin gel coating, fibrinogen from human plasma (Sigma-Aldrich) was dissolved in EBM-2 (Lonza) at 2 mg/mL and filter-sterilized. Polymerization of fibrinogen into fibrin was initiated by addition of 0.5 U/mL human thrombin (Sigma-Aldrich).

### Quantification of GF concentrations in hPL and hPLG-conditioned medium

The concentrations of VEGF-A, EGF, PDGF-BB and FGF-2 in three separate batches of hPLG preparations were determined by ELISA before gelation (i.e. without thrombin) using the Human Growth Factor II ELISA Strip Kit (Signosis, Santa Clara, US) according to the manufacturer’s instructions. GF release into basal medium by hPLG was also quantified by ELISA. hPLG was prepared as previously described in 12-well plates (1 mL/well) and, after one quick wash with PBS, 1 mL of medium was added immediately after polymerization. Samples of conditioned medium in the absence of cells were collected after 1, 3 or 5 days of incubation at 37 °C.

### Isolation of peripheral blood ECFCs

Peripheral blood was obtained by venepuncture from the median cubital vein of healthy drug-free volunteers. Informed consent was obtained from all donors. Donor consent procedure and venepuncture protocol were approved by the Ethics Committee of the University of Bath. Donor consent and blood collection were carried out in accordance with the guidelines approved by the Ethics Committee of the University of Bath. Isolation of ECFCs was performed as previously described^30^. Briefly, ECFCs were obtained from the peripheral blood mononuclear cell (PBMNC) fraction of whole human blood, which was separated by density gradient centrifugation using Ficoll-Paque PLUS (GE Healthcare, Uppsala, Sweden). PBMNCs were seeded at a density of 2 × 10^5^ cells/cm^2^ on tissue culture plastic coated with rat-tail collagen I (Corning, Bedford, US) in complete medium (EGM-2, Lonza), which contains 10% v/v FBS and supplier’s GF supplement (i.e. human basic Fibroblast Growth Factor, Vascular Endothelial Growth Factor, human Epidermal Growth Factor and long R3 Insulin-like Growth Factor 1) (Lonza cat. no. CC-4176). Plates were inspected for 3 weeks for colony appearance. Typical endothelial colonies appeared between days 14 and 21.

### Immunofluorescence

ECFCs were grown to confluence on either collagen I-coated plates or hPLG and fixed in 4% paraformaldehyde (PFA) for 15 min. Following fixation, cells were permeabilized in 0.1% Triton X-100 and blocked with 1% bovine serum albumin (BSA) for 30 min. Subsequently, the slides were incubated with anti-CD31 (Cell Signaling Technology, Danvers, US) or vWF primary antibody (Dako, Glostrup, Denmark) (1:200) overnight at 4 °C. After washing with PBS, slides were incubated with FITC Goat Anti-Mouse and FITC Goat Anti-Rabbit IgG (1:200) (Life Technologies, Carlsbad, US) for 1 h and Hoechst 33342 (Thermo Scientific, Rockford, US) (1 μg/mL). Epifluorescence images were obtained using a Leica DMI4000B microscope (Leica, Wetzlar, Germany), whereas confocal microscopy was performed using a LSM 510 META confocal microscope (Carl Zeiss AG, Jena, Germany).

### Cell proliferation

ECFCs were seeded at a density of 12,000 cells/well in 24-well plates on collagen I-coated wells or hPLG, and were cultured in complete medium (EGM-2, Lonza) with 10% FBS with or without pharmacological treatments (PD98059 50 μM, Ki8751 100 nM, Tivozanib 1 μM, Pazopanib 20 μg/mL). Cells were fixed after 24 h in 4% PFA and nuclei stained with Hoechst 33342 as above. Fluorescent images were acquired using a Leica DMI4000B microscope (objective: N Plan 10X/0.25) and nuclei were counted using ImageJ software 1.49 v (Wayne Rasband, National Institute of Health, US) to estimate cell numbers.

### Immunoblotting

ECFCs were lysed in radioimmunoprecipitation assay (RIPA) buffer in the presence of protease (Complete Ultra, Roche, Penzberg, Germany) and protein phosphatase inhibitors (sc-45045 and sc-45065, Santa Cruz Biotechnology, Santa Cruz, US). Proteins were separated by SDS-PAGE and immunolabelled using specific antibodies for ERK1/2 (sc-94, Santa Cruz Biotechnology) and phospho-ERK1/2 (9101S, Cell Signaling). Extracts from cell-free hPLG-coated wells were utilized as negative controls, which highlighted the presence of dephosphorylated ERK1/2 from platelets. This did not interfere with the analysis of ERK1/2 phosphorylation. Blots were stained with IRDye 800CW or IRDye 680RD secondary antibodies and scanned with a LI-COR Odyssey CLx infrared imaging system. Densitometric analyses were performed using Image Studio Lite v5.0.21 (LI-COR, Lincoln, US).

### RT-qPCR

ECFCs isolated from 3 different donors were cultured in triplicate on collagen I-coated plates or hPLG for 24 h followed by RNA extraction. Total RNA was extracted with TRIzol (Life Technologies, Carlsbad, US) and further purified with the RNeasy Kit (Qiagen, Hilden, Germany). Briefly, ECFCs were lysed by adding 1 mL of TRIzol and then mixed with 200 μL of chloroform before centrifugation at 12,000× *g* for 15 min. The aqueous phase was collected for RNA extraction using the RNeasy kit, following the manufacturer’s instructions. Quantitect reverse-transcription kit (Qiagen) was used to generate cDNA (equivalent to 250 ng total RNA). The mRNA gene expression of CD31, eNOS, VE-cadherin, vWF, CXCR4, SDF-1, Angiogenin, VEGF-A, VEGFR2, FGFR-1, PDGFR-β was carried out using 300 nM gene-specific primers (see Supplementary Table 1) and Power SYBR Green PCR Master Mix (Life Technologies). The amplification of a single PCR product was confirmed by melting curve analysis. Gene-specific mRNA levels were estimated by the 2^−ΔΔCt^ analysis and normalized against GAPDH levels to obtain relative changes in gene expression, as previously described^45^. The amplification of a single PCR product was confirmed by melting curve analysis.

### *In vitro* 3D vasculogenesis assay

Encapsulation of ECFCs was performed in three different gel matrices: collagen I, fibrin and hPLG. For collagen encapsulation, rat-tail collagen I (Corning) was diluted to a final concentration of 1.5 mg/mL and polymerized by neutralizing the pH using 1 M NaOH, as previously described^44^. For fibrin encapsulation, fibrinogen from human plasma (Sigma-Aldrich) was dissolved in EBM-2 (Lonza) at 2 mg/mL and filter-sterilized. Polymerization of fibrinogen into fibrin was initiated by addition of 0.5 U/mL human thrombin (Sigma-Aldrich). For encapsulation in hPLG, a 20% (v/v) platelet lysate solution was prepared in EBM-2 and the same amount of thrombin was added as for pure fibrin gels. Cells were mixed with the three gel precursors at a density of 2 × 10^5^ cells/mL and 70 μL of each cell suspension was added per well of a 96-well plate. Gelation was completed by incubation at 37 °C for 10 min. Cells were then cultured for 3 days in serum-free basal medium for hPLG and with complete medium (containing 10% FBS plus recombinant GFs specified in the cell culture method – i.e. hFGF2, VEGF, hEGF and R3-IGF1) for collagen and fibrin gels. Constructs were fixed in 4% PFA and then double stained with FITC-conjugated Ulex europaeus agglutinin (UEA) (1:100, O/N) and Hoechst 33342 (1 μg/mL, 15 min), for assessment of endothelial growth. For live cell imaging of lumenized networks in hPLG, cells were incubated with Calcein AM (10 μM, 1 h) and Hoechst 33342 (1 μg/mL, 15 min). Epifluorescence images were obtained using a Leica DMI4000B microscope (objective: N Plan 10X/0.25), whereas confocal microscopy was performed with a LSM 510 META confocal microscope (objective: EC Plan Neofluar 10X/0.30 M27). Total length of the capillary network in the different conditions was measured using ImageJ 1.49 v (Wayne Rasband, National Institute of Health, US).

### Aortic ring/ECFC co-culture assays

The aortic ring angiogenic assay developed by Nicosia *et al.*^46^ was modified. Briefly, aortas were harvested from 4 to 6-week-old Wistar rats. After removing the surrounding fibro-adipose tissues and rinsing with EBM-2 culture medium, the aortas were sectioned into 1 mm ring segments. Rings were serum starved for 24 h in EBM-2 supplemented with gentamicin (30 μg/mL) and amphotericin (15 ng/mL), then embedded in collagen I, fibrin or hPLG and cultured for 3 days in EBM-2 with 2% v/v FBS. Phase contrast images were acquired with an EVOS FL microscope (Thermo Fisher Scientific, Waltham. US), and sprouting (area and length) was quantified using ImageJ software. 2 × 10^5^ ECFCs/mL were encapsulated in hPLG surrounding the aortic rings. Differential staining of human and rat endothelial cells was performed by incubating constructs with FITC-UEA (1:100, O/N) and TRITC-conjugated Isolectin GS-IB4 (1:100, O/N), respectively. Epifluorescence images were obtained using a Leica DMI4000B microscope (objective: N Plan 10×/0.25).

### Scanning electron microscopy (SEM)

ECFCs were encapsulated in hPLG and cultured for 3 days. Encapsulated cells or fresh fibrin and hPLG samples were fixed with 2.5% (v/v) glutaraldehyde overnight at 4 °C, followed by post-fixation with 1% osmium tetroxide containing 1% potassium ferrocyanide for 1 h. Gels were extensively washed in PBS, stained in 1% aqueous uranyl acetate solution for 1 h in the dark and dehydrated in an acetone series: 50%, 70%, 90%, 95% for 10 min each time and then in 100% for 20 min. Subsequently, samples were dried by evaporation with hexamethyldisilazane and coated with gold using a Sputter Coater S150B system (Edwards). Images were captured with a JSM-6480LV Scanning Electron Microscope (JEOL, Japan).

### Transmission electron microscopy (TEM)

ECFCs were encapsulated in hPLG and cultured for 3 days, after which they were fixed with 2.5% (v/v) glutaraldehyde overnight at 4 °C, followed by post-fixation with 1% osmium tetroxide containing 1% potassium ferrocyanide for 1 h. Gels were extensively washed in PBS, stained in 1% aqueous uranyl acetate solution for 1 h in the dark and dehydrated in an acetone series: 50%, 70%, 90%, 95% for 10 min each time and then in 100% for 20 min. Next, samples were infiltrated with Spurrs Epoxy Resin by an acetone/resin series starting with 1:1 for 2 h, then in 100% overnight. The samples were then embedded in 100% resin and polymerized at 70 °C for 8 h. Electron micrographs of 100 nm sections were taken using a JEM-1200EX II transmission electron microscope (JEOL, Japan).

### Statistical analysis

Data are expressed throughout as mean ± standard error and presented using Prism 6 (GraphPad Software, La Jolla, US). Statistical significance of the difference between pairs of observations was performed using the non-parametric Mann-Whitney U-test. Statistical significance for multiple observations was analyzed by one-way ANOVA with Bonferroni post-test, after normal distribution and equal variance of the samples were confirmed using the Shapiro-Wilk normality test and Bartlett’s test for homoscedasticity, respectively.

## Additional Information

**How to cite this article**: Fortunato, T. M. *et al.* Platelet lysate gel and endothelial progenitors stimulate microvascular network formation *in vitro*: tissue engineering implications. *Sci. Rep.* **6**, 25326; doi: 10.1038/srep25326 (2016).

## Supplementary Material

Supplementary Information
