# Supplementary material for: Platelet lysate gel and endothelial progenitors stimulate microvascular network formation in vitro: tissue engineering implications
Source: Sci Rep. 2016 May 4;6:25326. doi: 10.1038/srep25326 (PMC4855184; doi:10.1038/srep25326)
Supplement: Supplementary Information [file srep25326-s1.pdf]

Platelet lysate gel and endothelial progenitors stimulate capillary formation *in vitro*: implications for tissue engineering.

Tiago Moderno Fortunato[1], Cristina Beltrami[2], Costanza Emanueli[2], Paul De Bank[1] and Giordano Pula[1]
[1]Department of Pharmacy and Pharmacology, University of Bath, Bath Spa (UK)
[2]Bristol Heart Institute, University of Bristol, Bristol (UK)
Corresponding author: G Pula, Department of Pharmacy and Pharmacology, University of Bath, Claverton Campus, Bath Spa (UK), BA2 7AY, Tel. +44 (0) 1225 385 755, Fax: +44 (0) 1225 386 114, email: G.Pula@bath.ac.uk

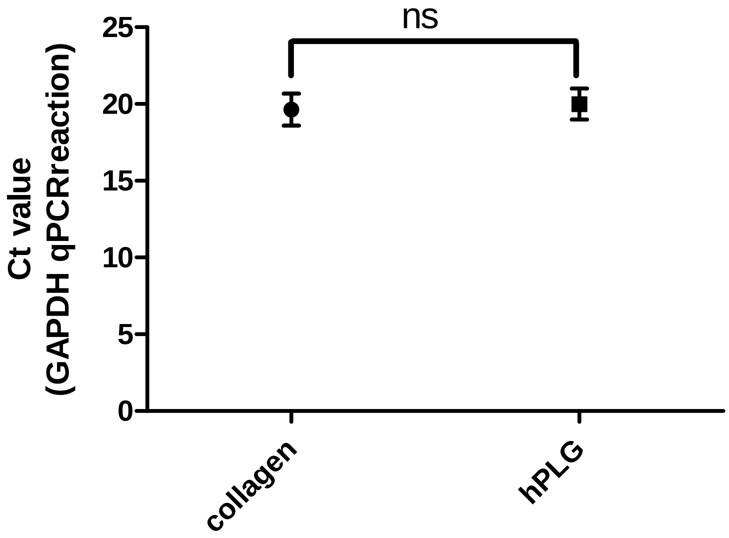

Supplementary Figure 1: GAPDH expression in ECFCs cultured on collagen or hPLG. Total RNA was extracted from ECFCs cultured on traditional coating (rat collagen) or hPLG for 24h and expression of selected genes was quantified by RT-qPCR. The amplification of a single PCR product was confirmed by melting curve analysis. Gene-specific mRNA levels were estimated by the 2-ΔΔCt analysis and normalized against GAPDH levels to obtain relative changes in gene expression, as previously described[1]. Data show mean±SEM of the Ct (cycle threshold) for collagen and hPLG cultures. Ct is defined as the number of cycles required for the fluorescent signal to cross the threshold. Ct levels are inversely proportional to the amount of target nucleic acid in the sample. No statistical difference was observed (Mann-Whitney U-test, n=3).

[1]Livak KJ, Schmittgen TD (2001) Analysis of relative gene expression data using real-time quantitative PCR and the 2(-Delta Delta C(T)) Method. Methods 25: 402-408.

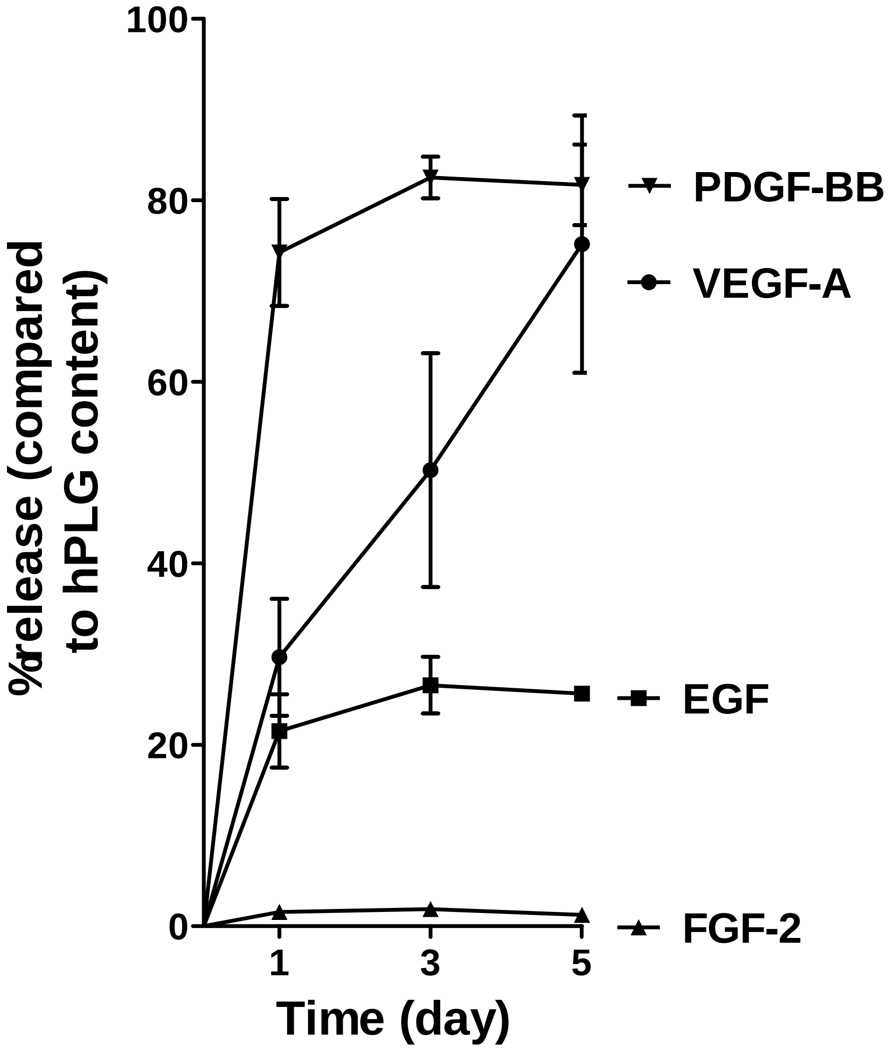

Supplementary Figure 2: Growth factor release expressed as % of hPLG content). Growth factor by hPLG in 1, 3 and 5 days of culture conditions were determined by ELISA using the Human Growth Factor II ELISA Strip Kit (Signosis, Santa Clara, US) according to the manufacturer's instructions. hPLG was prepared as previously described in 12-well plates (1ml/well). After one wash in PBS, 1mL of medium was added immediately after polymerization. Samples of conditioned medium in the absence of cells were collected after 1, 3 or 5 days of incubation at 37 ºC. Data show the release as % of the content in hPLG (shown in Figure 1C) and are presented as mean±SEM. Data are representative of three independent experiments.

Supplementary table 1. qPCR primer sequences

Target	Forward primer 5'-3'	Reverse primer 5'-3'
CD31	TCGGAAGGATAAAACGCGGTC	CCAAGGTGGGATCGTGAGG
eNOS	TGATGGCGAAGCGAGTGAAG	ACTCATCCATACACAGGACCC
VE-cadherin	AAGCGTGAGTCGCAAGAATG	TCTCCAGGTTTTCGCCAGTG
vWF	GCCCTGGTTGCCATTGTAATTC	AGCCTTGTGAAACTGAAGCAT
CXCR4	CCCACAATGCCAGTTAAGAAGA	ACTACACCGAGGAAATGGGCT
SDF-1	ATTCTCAACACTCCAAACTGTGC	ACTTTAGCTTCGGGTCAATGC
Angiogenin	CTGGGCGTTTTGTTGTTGGTC	GGTTTGGCATCATAGTGCTGG
VEGF-A	AGGGTCTCGATTGGATGGCA	AGGGCAGAATCATCACGAAGT
VEGFR-2	CCAGTGTCATTTCCGATCACTTT	GGCCCAATAATCAGAGTGGCA
FGFR-1	AATGAGTACGGCAGCATCAAC	ACCTCGATGTGCTTTAGCCAC
PDGFR-β	AGACACGGGAGAATACTTTTGC	AGTTCCTCGGCATCATTAGGG
GAPDH	AGCCGCATCTTCTTTTGCGT	TGACGAACATGGGGGCATCA

Supplementary table 2. Test of normality and homoscedasticity for data presented in Figures 3B, 3D, 3F, 4A and 7D

**Figure 3B**										
	Collagen:	Vehicle	Ki8751	Tivozanib	Pazopanib	hPLG:	Vehicle	Ki8751	Tivozanib	Pazopanib
		1.198925	0.8602151	0.937634	1.021505		2.770318	1.222615	0.8127208	1.399293
		1.021505	0.5322581	0.6827957	1.032258		2.29682	0.9469965	1.611307	1.498233
		1.478495	0.5537634	0.8655914	0.6344086		2.840989	0.9116608	1.533569	1.194346
		1.333333	0.7526882	0.8978494	1.05914		2.325088	1.053004	0.7985865	1.477032
		0.9301075	0.688172	0.7849463	0.9408602		2.59364	1.187279	1.378092	1.420495
		1.392473	0.6182796	0.7741935	0.8591398		2.763251	0.8339223	1.289046	1.75265
	**Variance =**	0.04661815	0.0156945	0.00880813	0.02556846		0.05625073	0.02432128	0.12457872	0.03263204
**Shapiro-Wilk test**	**W =**	**0.9417**	**0.9487**	**0.9602**	**0.8455**		**0.8527**	**0.9316**	**0.8619**	**0.9406**
	**Conclusion =**	**Data are normally distributed**								
**Bartlett's test**	**Chisquare**	**=**	**10.9661**							
	**P value**	**=**	**0.14**							
	**Conclusion**	**=**	**Data have equal variance**							

Figure 3D

	Collagen:	Vehicle	PD98059	hPLG:	Vehicle	PD98059
		1.198925	1.107527		2.770318	1.491166
		1.021505	1.134409		2.29682	1.830389
		1.478495	1.193548		2.840989	1.992933
		1.333333	1.198925		2.325088	1.65371
		0.9301075	0.9892473		2.59364	1.844523
		1.392473	0.9408602		2.763251	1.667845
	**Variance:**	0.04661815	0.01143196		0.05625073	0.031473346
**Shapiro-Wilk test**	**W =**	**0.9417**	**0.8907**		**0.8527**	**0.9688**
	**Conclusion =**	**Data are normally distributed**				
**Bartlett's test**	**Chisquare**	**=**	**2.9795**			
	**P value**	**=**	**0.395**			
	**Conclusion**	**=**	**Data have equal variance**			

Figure 3F

		Collagen	Collagen + hPL	Fibrin	Fibrin + hPL	hPLG
		1.015544	1.3057	1.158537	1.597561	2.227612
		1.212435	1	1.103659	1.615854	2.048508
		1.160622	1.222798	1.353659	1.420732	2.227612
		1.243523	1.53886	1.371951	1.371951	2.132463
		0.968912	0.9082902	1.25	1.347561	2.003731
		1.07772	0.880829	1.432927	1.445122	2.014926
	**Variance:**	0.01213901	0.06676302	0.01680048	0.01302426	0.010455753
**Shapiro-Wilk test**	**W =**	**0.9419**	**0.9200**	**0.9380**	**0.8697**	**0.8516**
	**Conclusion =**	**Data are normally distributed**				
**Bartlett's test**	**Chisquare**	**=**	**6.75543**			
	**P value**	**=**	**0.149**			
	**Conclusion**	**=**	**Data have equal variance**			

Figure 4A

		Collagen	Fibrin	hPLG
		895.62	1532.96	4715.09
		861.95	1903.07	4319.56
		1028.54	1777.7	4148.83
		1104.29	1846.04	4245.98
		927.02	1704.57	4100.35
		840.63	2012.66	4270.18
	**Variance:**	10576.0999	27758.7504	47840.4372
**Shapiro-Wilk test**	**W =**	**0.9074**	**0.9873**	**0.8292**
	**Conclusion =**	**Data are normally distributed**		
**Bartlett's test**	**Chisquare**	**=**	**2.50513**	
	**P value**	**=**	**0.286**	
	**Conclusion**	**=**	**Data have equal variance**	

Figure 7B

	Area:	Collagen	Fibrin	hPLG	Length:	Collagen	Fibrin	hPLG
		0.13	0.23	3.61		0.0375	0.0731	0.5741
		0.01	0.12	4.02		0.0102	0.0627	0.5869
		0.02	0.09	3.88		0.0440	0.0686	0.5488
		0.25	0.41	4.11		0.0562	0.0154	0.5461
		0.06	0.24	3.96		0.0254	0.0814	0.6114
		0.09	0.15	3.84		0.0378	0.0256	0.5975
	**Variance:**	0.00786667	0.01346667	0.02993987	**Variance:**	0.000250306	0.00073912	0.00069161
**Shapiro-Wilk test**	**W =**	**0.8970**	**0.9021**	**0.9639**	**W =**	**0.9692**	**0.8564**	**0.9321**
	**Conclusion =**	**Data are normally distributed**			**Conclusion =**	**Data are normally distributed**		
**Bartlett's test**	**Chisquare**	**=**	**2.17161**		**Chisquare**	**=**	**1.5247**	
	**P value**	**=**	**0.338**		**P value**	**=**	**0.467**	
	**Conclusion**	**=**	**Data have equal variance**		**Conclusion**	**=**	**Data have equal variance**	
